# Bilateral facial palsy as presenting symptom of post-transplant relapsed acute myeloid leukemia treated with venetoclax: a case report and literature review

**DOI:** 10.1007/s00277-025-06647-w

**Published:** 2025-10-27

**Authors:** Cecilia Diamanti, Vincenzo Apolito, Manuela Spadea, Valeria Ceolin, Anna Mussano, Alessio Tomatis, Marta Barone, Paola Quarello, Francesco Saglio, Franca Fagioli

**Affiliations:** 1 Department of Public Health and Pediatrics, Postgraduate School of Pediatrics, University of Turin, Turin, Italy; 2 Department of Pediatric Hematology and Oncology, Ospedale Infantile Regina Margherita, Turin, Italy; 3 Department of Public Health and Pediatrics, University of Turin, Turin, Italy; 4 Radiotherapy Unit, Ospedale Infantile Regina Margherita, Turin, Italy

**Keywords:** Acute myeloid leukemia, Extramedullary relapse, Facial palsy, HSCT, Venetoclax, Pediatrics

## Abstract

Extramedullary relapse (EMR) of acute myeloid leukemia (AML) after allogeneic hematopoietic stem cell transplantation (HSCT) is rare and challenging condition, associated with poor outcomes, especially in children. We describe a 5-year-old boy with therapy-related AML who developed bilateral facial palsy as the first sign of EMR, eight months after HSCT. Initial investigations, including bone marrow (BM), cerebrospinal fluid (CSF), and MRI, were inconclusive. Progressive symptoms and rising of minimal residual disease prompted repeated evaluations and, ultimately, led to the diagnosis of a bilateral extramedullary leukemic infiltration. The patient was treated with venetoclax combined with high-dose cytarabine and idarubicin, achieving complete resolution of the EMR and also first-ever molecular remission of the disease, further consolidated with a second HSCT. This case underscores the diagnostic challenges of EMR presenting with neurologic symptoms and supports the feasibility and efficacy of venetoclax-based intensive chemotherapy for EMR, even after prior HSCT, in pediatric AML patients.

**Supplementary Information:**

The online version contains supplementary material available at 10.1007/s00277-025-06647-w.

## Introduction

Prognosis of extramedullary relapse (EMR) of acute myeloid leukemia (AML) is poor, particularly after allogeneic hematopoietic stem cells transplant (HSCT) [1–4]. There is no clear consensus regarding the best approach in these patients, however venetoclax-based regimens demonstrated promising activity in relapsed/refractory (R/R) AML [5–7].

We herein report a case of EMR in a 5-year-old AML patient after HSCT who received venetoclax plus chemotherapy (high-dose cytarabine with idarubicin).

## Case description

In 2023, our patient, who had a history of nephroblastoma treated with radio-chemotherapy and right nephrectomy, was diagnosed with AML. Cytogenetic analysis revealed CBFβ/MYH11 (inv(16)) fusion transcript, which was quantified by Real-Time PCR (RT-PCR) (Table 1). As AML was considered therapy-related, the patient received two cycles of CPX-351, achieving complete remission (CR). However, given the availability of a sibling donor and the positivity of CBF/MYH11 on bone marrow (BM) at the end of treatment (0.165 copies/copies of ABL × 100), the patient proceeded to HSCT. Conditioning was based on busulfan, cyclophosphamide and melphalan. Three months after HSCT the patient was in CR with full-donor chimerism. RT-PCR showed a one-log reduction in CBF/MYH11 transcript compared to pre-transplant, but still remained positive in BM (0.0311) and peripheral blood (PB, 0.0742). No graft-versus-host disease (GVHD) occurred, thus cyclosporine was discontinued 160 days after HSCT.

**Table 1:** Evolution of disease as reflected by CBFβ/MYH11 transcript levels

Date	Material	Result
*17/02/2023*	BM	89.21
*16/02/2023*	BM	0.165
*13/06/2023*	BM	0.033
*28/07/2023*	BM	0.0174
*11/09/2023*	BM	0.0172
*08/11/2023*	BM/PB	0.0742/0.0311
*29/12/2023*	BM	0.4679
*02/01/2024*	BM	1.1669
*08/01/2024*	BM	0.8065
*19/01/2024*	BM	4.0636
*04/03/2024*	BM/PB	negative/negative
*03/04/2024*	BM/PB	negative/negative
*10/05/2024*	BM	negative

*BM* Bone Marrow, *PB* Peripheral Blood

Results are expressed as (copies of CBFb-MYH11A/copies of ABL) × 100

Eight months post-HSCT, the patient experienced otalgia and left-sided facial hemiparesis. Neurological and otolaryngological evaluations were performed, along with magnetic resonance imaging (MRI), which yielded negative results. Complete blood counts were normal, consequently the final diagnosis was peripheral hemiparesis of the facial nerve (Bell’s palsy), for which prednisone 1 mg/Kg was initiated. RT-PCR in peripheral blood showed one-log increase of CBF-MYH11 (0.4679). However, at the time, this was not considered sufficient to define a clear relapse and was not ultimately deemed related to the symptom.

A few days later the patient was re-examined, as the paresis had progressed to the opposite side: at that time, BM and cerebrospinal fluid (CF) were negative for blasts and the patient presented stable full-donor chimerism. However, RT-PCR showed further increased CBF-MYH11 transcript, both in PB (1.1669) and BM (0.8065).

**Fig. 1:**
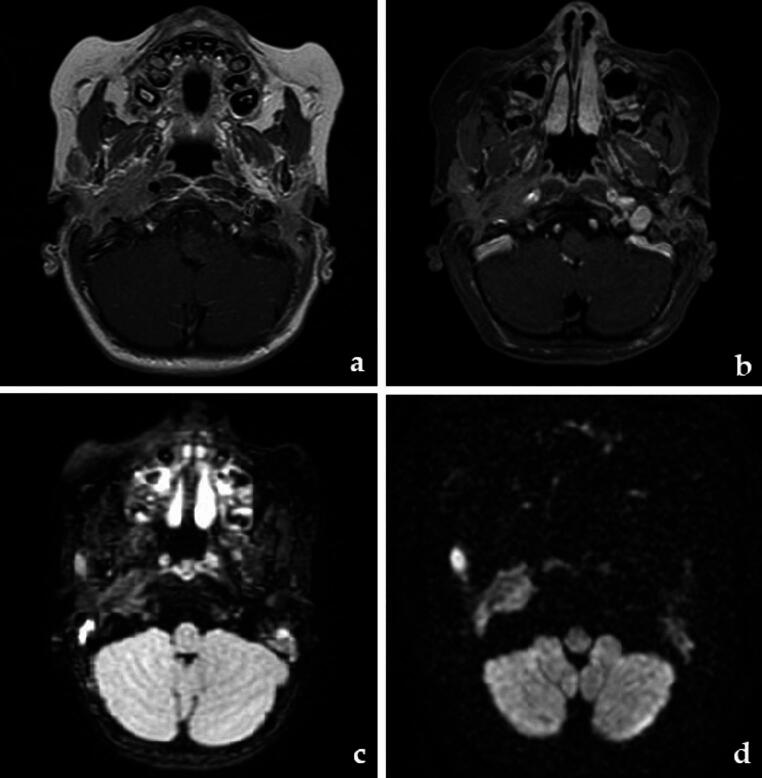
MRI scans of the iEMR. **a**) T1, **b**) T1 with contrast enhancement, **c**) FLAIR, **d**) DWI

Simultaneously, the patient developed otitis media. Otolaryngological and neurologic evaluations raised the suspicion of an infectious/inflammatory etiology of the paralysis. However, a second MRI was repeated two weeks after the first one and showed a bilateral extramedullary mass infiltrating the stylomastoid foramen (right: 35 × 14 mm, left: 24 × 11 mm, Fig. 1). A local biopsy confirmed the presence of leukemic cells, while BM and CF still returned negative. At this point, CBF-MYH11 transcript in BM was significantly elevated (4.0636).

Subsequently, our patient received venetoclax 360 mg/m^2^ in combination with intravenous cytarabine (1000 mg/m^2^ BID in 4 days) and a single dose of intravenous idarubicin (12 mg/m^2^). Venetoclax was administered for 19 days, but discontinued thereafter due to mucositis, which impaired oral intake, and the profound myelosuppression achieved. After the first cycle MRI showed complete resolution of the lesion. For the first time, RT-PCR on BM was negative for CBF-MYH11.

Afterward, the patient received two consolidation cycles with cytarabine and venetoclax. Each cycle was well tolerated, without significant complications. During treatment, antifungal prophylaxis with liposomal amphotericin B was administered twice weekly. Afterward, a second HSCT from a matched-unrelated donor was scheduled as consolidation strategy. Conditioning was based on etoposide plus total marrow and lymphoid irradiation to achieve better local disease control.

**Fig. 2:**
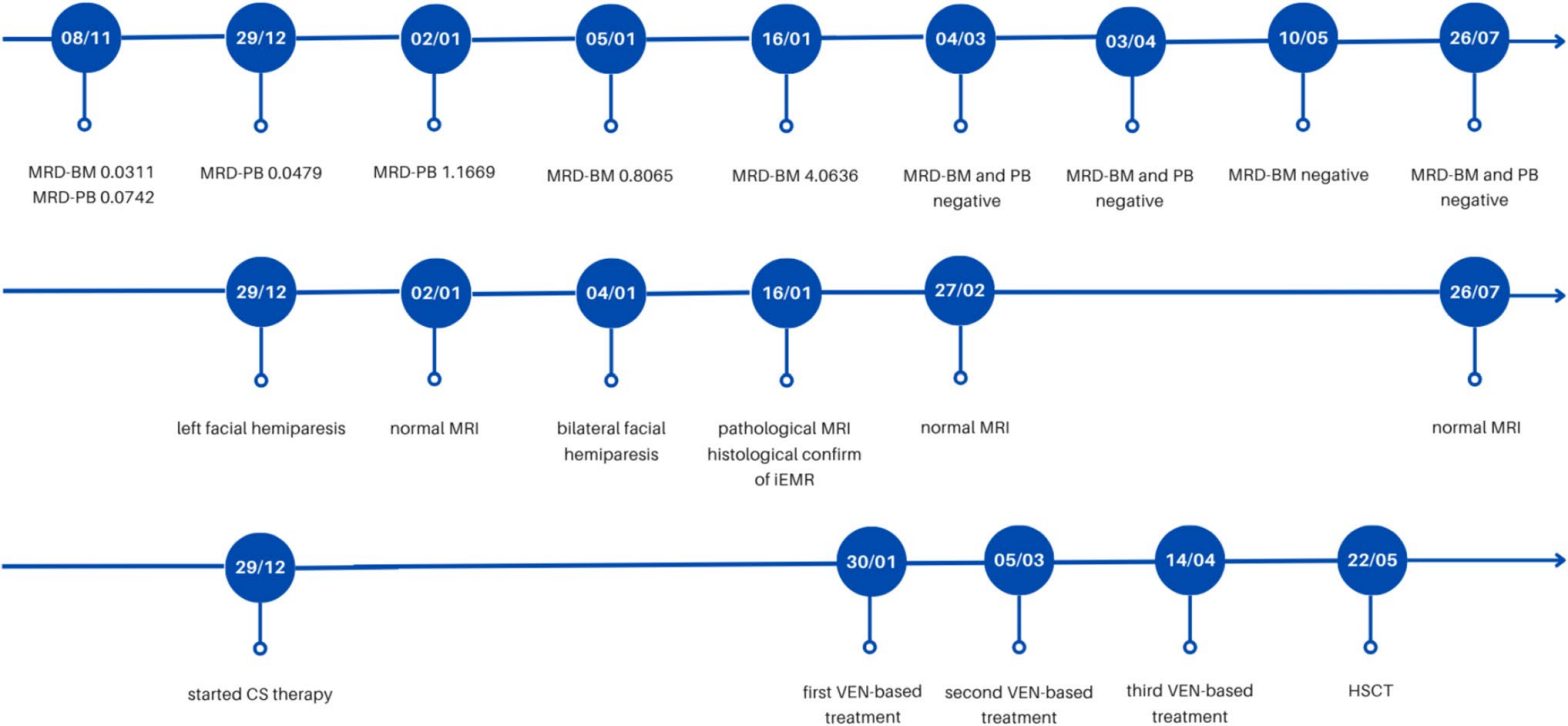
Timeline of key clinical events (year 2023/2024). MRD: Minimal Residual Disease; BM: Bone Marrow; PB: Peripheral blood; CS: Corticosteroid; MRI: Magnetic Resonance Imaging; iEMR: isolated Extramedullary Relapse; VEN: venetoclax; HSCT: Hematopoietic Stem Cell Transplant

The patient subsequently developed an acute cutaneous GVHD grade IV, and received steroids, ruxolitinib and extracorporeal photopheresis. Currently, 12 months after HSCT and 16 months after EMR, the patient is alive, in CR, with CBF/MYH11 negative and no EMR (Fig. 2).

## Discussion

This case report presents several noteworthy clinical and diagnostic features that warrant discussion.

First, although rare, facial palsy can be a challenging sign of AML relapse and it should be thoroughly investigated, since delayed diagnosis is common and it may have significant consequences [1–3].

In our case, bilateral facial paralysis was not initially associated with clear signs of relapse on the first MRI, BM or PL, but the increased CBF/MYH11 transcript raised suspicion of disease recurrence, prompting us to reevaluate the patient several times, despite the initial negative results.

We conducted a literature review and identified 12 cases of pediatric AML presenting with uni- or bilateral facial palsy (either at initial diagnosis or upon relapse) reported between 2014 and 2024 (Table 2, next page) [8–18]. A considerable number of these cases were initially misdiagnosed, leading to inappropriate treatments with steroids and delaying the correct diagnosis for more than 2 months in some instances. To note, most of the patients reported, had CBF AML, as our case.

**Table 2:** Pediatric cases of AML presenting with uni- or bilateral facial palsy reported between 2014 and 2024

Case	Age	Sex	Onset/relapse	PB	BM	FAB	Translocation	CNS involvement	Imaging	Misdiagnosis	Interval since diagnosis	Other treatment	Treatment	Outcome
1^8^	1.7	F	relapse	NA	yes (80%)	NA	t(16;16), CBFA2T3-Glis2	NA	NA	NA	NA	NA	ChT	death
2^9^	6	M	onset	no	yes (7%)	NA	t(8;21)	no	pathological (CT)	yes (complications of acute otitis media)	> 2 months	oral steroid, antibiotics	ChT	progression
3^10^	11	M	relapse	no	yes	NA	NA	no	pathological (MRI)	yes (complications of acute otitis media)	> 2 weeks	oral steroid, antibiotics	None	abandoned
4^11^	0.8	M	onset	no	yes	M5	NA	yes	initially normal, then pathologi	yes (bacterial meningitis)	> 2 months	oral steroid, antibiotics	ChT	abandoned
5^12^	1	M	onset	yes	yes	M1	trisomy 8, t(14;20)	no	suggestive	yes (acute otitis media)	> 1 month	antibiotics, tympanotomy	ChT	recovered
6^13^	10	F	relapse	no	no, then yes	M4	NA	no, then yes	initially normal CT, then pathological (MRI)	yes (Bell’s pasly)	rapid	oral steroid	ChT	BM relapse and death
7^13^	2	M	relapse	yes	yes	M5	NA	no	pathological MRI	no	rapid	-	NA	progression and death
8^14^	14	M	onset	yes	yes	NA	t(8;21)	no	normal MRI	no	rapid	-	ChT	NA
9^15^	2.6	F	onset	yes	yes	NA	trisomy 6, t(8;21)	no	pathological CT	yes	4 days	oral steroid	ChT	recovered
10^16^	8	M	relapse	no	no	M2	t(8;21)	no	pathological MRI	yes (Bell’s palsy)	NA	oral steroid	ChT	progression and death
11^17^	13	F	onset	yes	yes	NA	t(8;21)	no	pathological CT	no	> 2 months	-	ChT	recovered
12^18^	4	F	onset	yes	yes	NA	t(8;21)	NA	pathological MRI	yes (Bell’s palsy)	5 days	oral steroid, antiviral	ChT	recovered
This case	5	M	relapse	no	no	M4	inv(16)	no	initially reported negative, then pathological MRI	yes (Bell’s palsy)	3 weeks	oral steroid	VEN + ChT	recovered

*NA* Not Applicable; CT: Computer Tomography, *MRI*: Magnetic Resonance Imaging, *ChT* Chemotherapy

Second, our case also highlights the importance of quantitative minimal residual disease (MRD) monitoring: in our patient, increased CBF-MYH11 transcript was the first and only sign raising concern for AML relapse, prompting a rapid reassessment. Moreover, as suggested by guidelines primarily developed by adult hematologists [19], the CBF transcript can be assessed not only in BM but also in PB, yielding comparable results, as observed in our case. This approach significantly simplifies monitoring in the pediatric population. Furthermore, our patient did not achieve MRD negativity either before or after the first HSCT, a condition that significantly increases the risk of relapse, as occurred in our case.

Third, to date, no consensus has yet emerged on the best strategy for these patients [5–7]. In the adult setting, venetoclax demonstrated to be effective in AML, initially in combination with hypomethylating agents (HMAs) [6, 7, 20–26], and later with intensive chemotherapy [7, 22, 27–31]. In a phase 1 trial conducted in children with R/R AML, CR rate was 70% with venetoclax plus cytarabine and idarubicin. However, EMR were not included [30].

Research on EMR and venetoclax is scarce [23–25]: most available data pertains to adults, consisting in a few case reports [6, 23] and retrospective studies [23–25], which focused mostly on the combination with HMAs. In one of the largest cohorts of AML children receiving venetoclax, only two had EMR after HSCT, but both were treated with HMAs (one did not respond) [8]. CR in the whole cohort was 51.6%. Cousson et al. retrospectively analyzed a cohort of 12 pediatric patients with R/R AML treated with venetoclax-based regimens: of those, 5 presented with extramedullary disease (4 CNS and 1 skin). One patient achieved CR with venetoclax plus CT, but subsequentely died after HSCT due to a fungal infection [32].

Due to the limited pediatric data on venetoclax dosing, in our case the standard recommended dosage was administered. To avoid the well-documented CYP3A-mediated interaction with azole antifungals, with altered venetoclax plasma levels, antifungal prophylaxis was performed with liposomal amphotericin B twice weekly.

Beyond the peculiarity of facial palsy, to the best of our knowledge, this is the first pediatric report of EMR after HSCT treated with venetoclax plus high-dose chemotherapy. In this setting there are no comparative data to guide the choice of the best therapy to associate with venetoclax. However, in our case, chemotherapy plus venetoclax was clearly effective, leading to the achievement of MRD negativity, which had never been attained with previous therapies. This is of utmost importance, since the patient still experiences persistent CR at 12 months from HSCT.

Moreover, the combination therapy was well tolerated by our patient despite a substantial burden of prior treatments, including a HSCT, chemoradiotherapy, and nephrectomy. This allowed to proceed to a second HSCT, as consolidation strategy, as the child was fit and had not experience serious complications.

In conclusion, this case report, alongside a review of the existing literature, underscores the diagnostic challenges associated with facial palsy as a rare manifestation of AML relapse. It emphasizes the critical importance of early recognition and thorough investigation in such atypical presentations. Additionally, it provides further evidence supporting the use of venetoclax in combination with chemotherapy in case of extramedullary involvement in AML, even after HSCT. Ongoing and future investigations will be crucial to more precisely define the optimal integration of venetoclax into the therapeutic armamentarium for pediatric AML.

## Electronic Supplementary Material

Below is the link to the electronic supplementary material.

Supplementary Material 1

## Data Availability

Open access funding provided by Università degli Studi di Torino within the CRUI-CARE Agreement

## Abbreviations

EMR: Extramedullary relapse
AML: Acute myeloid leukemia
HSCT: Hematopoietic stem cell transplantation
R/R: Relapsed/refractory
RT-PCR: Minimal residual disease
CR: Complete remission
BM: Bone marrow
PB: Peripheral blood
GVHD: Graft versus host disease
MRI: Magnetic resonance imaging
CSF: Cerebrospinal fluid
MRD: Minimal residual disease
HMAs: Hypomethylating agents
