# Supplementary figures and images for: Bilateral facial palsy as presenting symptom of post-transplant relapsed acute myeloid leukemia treated with venetoclax: a case report and literature review
Source: Ann Hematol. 2025 Oct 27;104(12):6427–33. doi: 10.1007/s00277-025-06647-w (PMC12764545; doi:10.1007/s00277-025-06647-w)

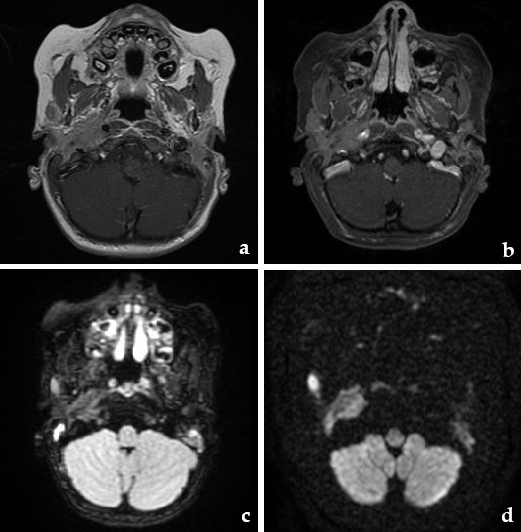

Supplement: Supplementary file 1 — Supplementary Material 1 [file 277_2025_6647_MOESM1_ESM.png]
